# Strategic Choices for Social Responsibility of Startups in China

**DOI:** 10.3389/fpsyg.2021.719454

**Published:** 2021-09-27

**Authors:** Bojing Liu, Lu Lu, Hua Zhang, Chanjuan Liu

**Affiliations:** ^1^School of International Studies, Wenzhou Medical University, Wenzhou, China; ^2^School of Innovation and Entrepreneurship Education, Wenzhou Medical University, Wenzhou, China; ^3^School of Foreign Languages Studies, Wenzhou Medical University, Wenzhou, China

**Keywords:** normalization, social responsibility, startups, SCP analytical framework, SCR model, COVID-19 pandemic

## Abstract

This study uses the structure–conduct–performance analytical framework in industrial organization theory to analyze Chinese startups’ corporate social responsibility (CSR) assuming normalization after the COVID-19 pandemic. Specifically, we take the external impact of the pandemic on startups during the pandemic as a starting point for analyzing the changes in the structure of startups and their CSR performance. We find a positive correlation between the pandemic and the performance of startups. We propose that the CSR of startups is not simply altruism but must involve an “altruistic and self-interested” mechanism. Therefore, this study proposes that during the pandemic, startups need to rebuild their CSR model. Furthermore, the company’s “economic man” and “social man” are interdependent; economic, ethical, and legal responsibilities are parallel and charitable responsibilities remain the highest pursuit amid the pandemic. The social responsibility of startups as the COVID-19 pandemic normalizes is a strategic choice.

## Introduction

The coronavirus disease (COVID-19) pandemic has had an unprecedented “butterfly effect” on the international order, national economies, all walks of life, and individual lives. The pandemic has also caused tremendous changes in consumption patterns and unexpected external shocks to startups, forcing them to focus on corporate culture, corporate philosophy, production methods, sales, service, and so on. The pandemic has had a far-reaching impact on the production and operational activities of startups. For a startup, whether passively driven by external pressure or an active strategic choice for internal transformation, the social responsibility it assumes has become a new mission for the times and an inevitable historical choice for the startup to develop in the context of the pandemic. During this period of pandemic normalization, prevention and control regulations will be strictly observed and the human population will be able to return to a pre-outbreak way of life. Therefore, in the context of the pandemic, this study applies the structure–conduct–performance (SCP) analytical framework from industrial organization theory to analyze startups’ performance of corporate social responsibility (CSR), the impact of the pandemic on industrial structure, the behavioral response of startups, and the fulfillment of social responsibilities. For the four dimensions of changes brought about by CSR performance, this study conducts an in-depth analysis of the evolutionary characteristics and trends of CSR, and on this basis, reconstructs the CSR model against the backdrop of normalization after the COVID-19 pandemic. We analyze the reconstruction path of CSR in the era of the COVID-19 pandemic and provide a reference for new startups and their social responsibility in countries around the world that are on the verge of normalizing after the epidemic.

## Materials and Methods

### Applying the Structure–Conduct–Performance Analytical Method to Corporate Social Responsibility

**FIGURE 1:**
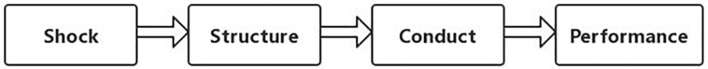
SCP analytical paradigm.

The COVID-19 pandemic has had a huge, irregular, and sudden external impact on startups and their operations. The pandemic has also allowed startups to shoulder a greater share of social responsibility. The SCP model is mainly used to analyze the strategic adjustment and behavior change of enterprises when an industry or enterprise is subject to external shocks. To systematically observe and study the development characteristics and social responsibility trends of startups during the pandemic, this study adopts the SCP analytical method (Figure 1). Based on an analysis of the pressure that startups face in fulfilling their social responsibilities, the *status quo* of startups’ social responsibility, and the behavioral responses of startups, this study conducts a standardized and three-dimensional analysis of the social responsibility phenomenon of startups during the normalization phase of the COVID-19 pandemic.

### The Structure–Conduct–Performance Analytical Method

The SCP analytical method was established in the 1930s by the Harvard industrial economics expert Joe S. Bain, among others (Mueller and Raunig, 1999). This method provides a framework for an industrial analysis of market structure, behavior, and performance that can investigate specific links based on systematic logic (Panagiotou, 2006). Under the SCP framework, the market structure determines the behavior of the company in the market, and the behavior of the company determines the market’s economic performance in all respects (Hopkins and Woodward, 1965). The SCP analytical method can intuitively reflect the changes caused by the pandemic in the entire startup market structure, which leads to changes in market behavior and market performance, and provides a reference for companies to formulate their own strategies and operational changes (Niu and Zhang, 2010). Therefore, this study adopts the SCP analytical model, starting with the dynamic balance of the industrial structure of startups and corporate behavior, that is, possible strategic adjustments and behavioral changes (Clarkson and Miller, 1982). The resulting market encompasses multiple dimensions, such as the effectiveness of resource allocation and business performance. Based on the establishment of a new model for startups to perform their social responsibility during the pandemic, we systematically observe and study the development trends and characteristics of startups’ social responsibility to better guide the formation of industrial policies, determine industry development trends, and inspire the selection of corporate strategies.

### External Impact of Startups Under the Structure–Conduct–Performance Framework

**FIGURE 2:**
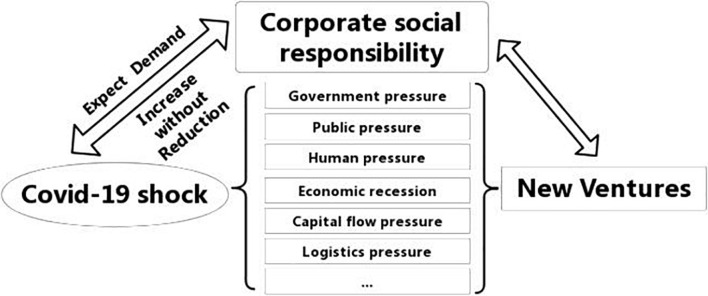
External impact on the social responsibility of startups during the pandemic.

As shown in Figure 2, the pandemic has impacted startups in all aspects of the fulfillment of their social responsibilities. The latest World Economic Outlook report, issued by the International Monetary Fund on October 13, 2020, shows that the world economy is expected to shrink by 4.4% in 2020. Although this is slightly better than the forecast in June 2020, the world economy remains in a deep recession (Jin, 2020), industrial and supply chains are hindered, international trade and investment are shrinking, the commodity market is in turmoil, and the macro environment for the sustainable development of the global economy has sharply deteriorated (Altig et al., 2020). Even in Asian countries that are less affected by the pandemic than other parts of Asia or other regions, the economic shock caused by the pandemic has been more severe than the shocks from the global financial crisis of 2007–2008 (Han and Han, 2020). The COVID-19 pandemic first hit the entrepreneurial ecosystem in China, according to Start-up Genome, a market research firm. Within the first 2 months of the outbreak, financing for Chinese startups fell by more than 50%. In early 2020, the China Association of Small and Medium Enterprises conducted a survey on small and medium-sized enterprises and startups in China, involving 6422 enterprises. The data showed that 86.46% of these enterprises were greatly affected by the epidemic.

The position of startups’ social responsibility in their business decision-making, as a non-market strategy for startups, has also been greatly affected. Startups that are at an early stage of entering the market have multiple dimensions, such as corporate strategic decision-making and corporate operating philosophy. They have suffered a major hit, making it even more difficult for startups that were originally at the bottom of the industrial chain to fulfill their CSR. Some startups gradually perished during the pandemic, although some have been able to survive. Startups have also faced tremendous pressure during the pandemic. Within this context, the question has arisen of how they can complete their transformation into sustainable businesses during the pandemic. The difficulty for every startup is that among the many challenges they face, they need to build a more complete corporate ecosystem, transform the challenges into opportunities, and fulfill their social responsibilities. At the same time, at a micro level, the strict prevention and control measures adopted after the outbreak of the pandemic have cut off the flow of people and logistics to some extent, blocking the flow of labor, and disrupting supply chains, normal consumer services, and production in whole or part. The resumption of work and production has introduced tension to the capital flow of startups (Carroll, 1991b). The impact of the epidemic on startups is reflected in the following main aspects, among others: slowing market demand, supply chain disruption, and seriously insufficient corporate cash flow capacity. According to the Assessment Report on Impact of Covid-19 Pandemic on Chinese Enterprises issued by the United Nations Development Program (UNDP) in China, only 30% of the enterprises surveyed had cash flow to last for within 1 month, while less than 10% had cash flow to last 6 months or more. Due to the small scale of startups, the overall duration for which cash flow was expected to last would be lower than that of large and medium-sized enterprises. Furthermore, the procurement activities of domestic startups in their own countries and other countries have been seriously affected, especially in industries with a high degree of globalization and segmentation of supply chain, such as automobile manufacturing and textiles.

The pressure of the flow of people, logistics, and capital has made the relationships among startups and key stakeholders, such as employees, consumers, suppliers, and distributors, extremely tense and abnormal. This is critical for the management of startups and all their activities. Startup teams and capital investors have been affected to varying degrees. During the pandemic, startups have faced non-market shocks in fulfilling their social responsibilities, such as policy pressure and public opinion pressure (Huang and Ye, 2021). At the same time, some high-tech startups have made good use of their own advantages and characteristics and constructed a business operation model based on the core values of the company, which, to a certain extent, has reduced the impact of the pandemic on startups. However, some labor-intensive startups that rely on human resources in their main operating model have encountered difficulties in effectively completing changes in their business strategies during the pandemic, and there is limited room to talk about fulfilling CSR. Among the tertiary industries, tourism and catering have been the most seriously affected. During the pandemic, there has been a continuous increase in the expectations of governments and the public for startups to take on social responsibilities. Startups that aim to have a breakthrough amid such external shocks find it difficult to fulfill their social responsibilities.

### Changes Faced by Startups Due to the Pandemic

#### Changes in the Working Environment of Startup Teams

The pandemic caused major changes in the working environment of entrepreneurial startup teams in three main ways. First, the original workplace has changed. Because of the need to effectively control the spread of the pandemic, most startup companies require their entrepreneurial teams to work from home or a remote office. According to the Assessment Report on Impact of Covid-19 Pandemic on Chinese Enterprises released by the UNDP in China, working online from home has increased by 537%. This new working environment has reduced the enthusiasm of entrepreneurial teams to a certain extent and weakened effective communication and exchanges between various parts of these teams. Group cultural activities that can be organized under normal conditions cannot take place. Although communication and operating costs have been somewhat reduced, a sense of alienation has arisen between entrepreneurial teams, and the close working relationship between various departments has been interrupted.

Second, organization managers need to re-establish and construct an enterprise’s entrepreneurial philosophy and operating model. The pandemic has prompted changes in many industries. The rise of unstaffed factories and intelligent factories has changed the traditional relationships between people. A mutual exchange of work models has prompted managers to rethink corporate positioning in the context of the pandemic and better fit the changes in the external work environment. The business strategies of startups mostly depend on current market demand, and they tend not to consider medium- and long-term development. In this context, enterprises can take advantage of the pandemic to carry out medium and long-term development.

Third, the core team of startups must reconsider their employees’ future development (Ko et al., 2019). The rise of various types of high-tech companies during the pandemic has impacted traditional human-based enterprises. Within startups, the pace of “artificial intelligence” replacing “traditional labor” is accelerating, prompting entrepreneurial teams to plan ahead for the future development of employees and to plan each employee’s career.

#### Changes in the Degree of “Trust” Between Startups and Consumers

The pandemic has caused two different changes in startups’ responsibility to consumers. First, the pandemic has increased the requirement for delivering goods or services without direct physical contact between firms and their customers; some services using mutual contact as the main method can no longer do so, which might have increased consumers’ distrust in the company and its products. In some industries, sales and after-sales services have been interrupted because of the pandemic, in addition to such problems as failure to deliver and pay on time. Consumer complaints and rights protection incidents have increased significantly, and the public has become more skeptical about the external performance of CSR. This has led some smaller companies to be stuck in this “dilemma” caused by the pandemic and to face huge stress.

Second, some startups whose main products are networks and online platforms have gained public recognition during the pandemic. The particularity of their products and their forms enables these startups to better handle the pandemic and the relationship with external stakeholders, such as partners and competitors. This has directly changed the original operating status of startups, enhanced the degree of mutual “trust” with consumers, and enabled the corporate culture to achieve vertical and horizontal expansion during the pandemic, such that the corporate philosophy is rooted more deeply in the hearts of people. In addition, startups have found new opportunities to support their long-term sustainable development (Acs and Amorós, 2008).

#### Change in the Sense of Responsibility by Startups’ Core Entrepreneurial Teams

After the outbreak of the pandemic, startups’ core entrepreneurial team had a sense of responsibility in which pressure and opportunities began to coexist. First, during the pandemic, traditional handcrafts and labor-intensive industries were severely affected and required the core teams in the industrial chain to exert leadership to help upstream and downstream startups survive the difficult period, especially those that had only recently been established. It was especially important for firms to adjust quickly to correctly reflect the market and to complete the development and transformation of enterprises in their initial stages. Second, after the outbreak of the pandemic in China, major startups actively participated in combating the pandemic by donating money and materials, organizing volunteers, etc., even those in the early stages. Their collection of funds and use of external resources earned startups acclaim from the public, consumers, and the market (Moon et al., 2014). In this way, the startup established a corporate image and showed the company’s face. However, the core team still needed to attend to the fact that, despite the effects of the pandemic on the production and operation of startups, leading to a sharp drop in revenue, no corresponding reduction occurred in the government’s requirements for environmental governance. Startups have environmental responsibilities that put a certain amount of pressure on their investment.

At the same time, since the outbreak of the pandemic, the public and consumers have paid more attention to environmental protection, health, and other product characteristics, and sustainable consumption has become the mainstream in consumption, which in turn has led to some degree of environmental protection and transformation of startups. To take advantage of opportunities, startups need to reconsider the effectiveness, sustainability, and effectiveness of the product (Fregnan et al., 2020).

## Results

### The Choice and Performance of Social Responsibility by Startups During the Pandemic

#### Responsible Behavior Choices of Startups During the Pandemic

Because of the pandemic, startups have changed how they fulfill their social responsibilities. Based on the four classifications of Carroll’s social responsibility for startups, this study analyzes the performance of startups’ social responsibility.

### Economic Responsibility

Economic responsibility refers to the fact that as a component of the basic economic unit of society, an enterprise needs to produce products that consumers and society demand and provide corresponding services to earn profits. In the process of responding to the interference and impact of a series of external social factors caused by the pandemic, the primary economic responsibilities that enterprises need to bear are how to maintain the normal operation of the enterprise itself, especially in the case of employees working remotely, to maintain the production of the enterprise and the ability to serve society and consumers. At the beginning of the pandemic, of the 6422 small and medium-sized enterprises and startups in China, more than 70% took the initiative to produce and save: 47.24% were striving for part of the construction; 32.73% of enterprises planned to ramp up publicity and marketing; and 29.1% of enterprises were speeding up the transformation to online and digital transformation.

At a micro level, internet companies provide different types of companies with more mature remote office security services, which effectively stabilizes the *status quo* of corporate economic operations while optimizing collaborative office scenarios. It ensures the coordination of work between employees and the vitality of the enterprise itself, and minimizes the negative impact of the pandemic on the enterprise. However, many startups have not been able to rely on remote working for completing all aspects of production, sales, and after-sales, and thus, it has become difficult for them to continue to perform their social and economic responsibilities. Although the Chinese government introduced a series of related support policies for these startups, such as tax and rent reductions, targeted technical support, and a supply of personnel who resumed work and production, a large number of startups did not survive this difficult period of the pandemic. At the macro level, the global spread of the pandemic led to intermittent disruptions to imports and exports by various countries. Many startups that rely on imports and exports could not maintain sales during the pandemic and therefore, had to reduce production, undermining their continued development, thereby reducing employees’ enthusiasm for production.

### Legal Responsibility

Legal responsibility means that the company must abide by the laws and regulations of the state and local governments and conduct business activities to earn profits only within the scope permitted by the law (Liang and Renneboog, 2013). During the early period of the pandemic, various ordinary medical supplies, such as masks and protective equipment, became hot commodities, and some platforms and merchants engaged in misleading advertising of these goods, which drove up prices. At the time, most Chinese startups effectively fulfilled their social legal responsibilities, abiding by the laws and regulations of the country, and established a good public image of startups for two main reasons. First, the startups were in their initial stage of establishment, most of the core entrepreneurial teams were basically aware of the laws, and the companies had legal representation, which greatly reduced the chance that firms lacked market experience and exploited legal loopholes during the pandemic. Second, the Chinese government responded quickly to market fluctuations, formulated and promulgated relevant laws and regulations, stabilized the external market environment across the economy, imposed penalties on companies that violated laws, and cracked down on criminal activities. The process of growing wealth through legal means positively impacted the supply of pandemic prevention and control materials.

### Ethical Responsibility

Ethical responsibility means that companies must abide by existing norms and guidelines and clearly define their business ethics. However, some corporate behavior is outside legal norms and is not strictly illegal. Only startup behaviors expected by the public can be considered ethically responsible.

After implementing a lockdown in Wuhan, Hubei province, China announced the closure of the Lihan Channel and the suspension of public transport in the city, effectively closing down the city. Medical staff on the front lines of the pandemic encountered difficulties in obtaining food, transport, and housing. For example, SF Corporation organized a team of volunteers to shuttle medical staff back and forth every day to solve the medical transportation problem. The material needs of medical institutions and citizens were guaranteed. Although SF Express is not a startup, among the volunteer service teams the company organized, nearly half of the volunteers were from various startups or teams. During the pandemic, various types of startups from all over China rushed to Wuhan in different ways, providing a strong logistical guarantee for the transport of front-line medical supplies and supplies for daily life. Through internet connectivity, the effective mobilization of social resources, and the strong support of the logistics and transportation system, the impacts of the initial absence of government emergency resources and the delayed operation of non-governmental organizations were minimized. Startups met their ethical and other responsibilities through practical actions. Brand behaviors were in line with mainstream public opinion about national pandemic prevention, and thus, gave startups a good reputation. For example, the Chinese unicorn startup e-point rent, despite being hit by the pandemic, provided free IT services to customers across an industry to actively support pandemic-hit areas, and was seen to actively meet its social responsibility and become a role model for startups in the pandemic.

### Philanthropic Responsibility

Charitable responsibility is also called discretionary responsibility, which mainly refers to the willingness of enterprises to participate actively in improving overall social welfare. Charitable responsibility is not compulsory, and follows a company’s wishes, as perceived and evaluated by individual consumers (Wulfson, 2001). After the outbreak of the COVID-19 pandemic, Baidu donated 2 billion yuan, and Alibaba and General Technology donated more than 1 billion yuan. Compared with these industry leaders, most companies do not have the ability to donate such large amounts of money; however, this does not relieve the enterprise from its obligations for social responsibility.

Following the example of industry giants, startups that successfully fulfilled their philanthropic and social responsibilities injected a philanthropical core into their corporate culture. This reflects the core entrepreneurial team’s deep understanding of their responsibilities and opportunities in the COVID-19 era, and it is the most intuitive external embodiment of its entrepreneurial philosophy and core corporate values (Iwannanda et al., 2017). Therefore, the philanthropic responsibility undertaken by startups is not simple altruism but a mechanism that combines “altruism and self-interest.” The charitable responsibility and business behavior of enterprises are not two parallel modes; the full realization of the in-depth integration of political, economic, and social dimensions can better shape startups with self-supporting functions.

### Startups’ Performance of Social Responsibility During the Pandemic

A literature review (see Table 1) indicates that most scholars view social responsibility by enterprises as a key factor influencing consumer behavior. Many empirical studies show that customers’ evaluations of companies (e.g., startups or brand image) are usually based on startups and brands. The social strategy of startups is the fulfillment of their social responsibilities (Dacin, 1997). Under the same circumstances, startups with more social behavior can more easily convey brand symbols and brand differentiation values to consumers, because of the added value of their social responsibilities, and enterprises are more likely to be associated with consumers (Bhattacharya, 1999).

**TABLE 1:** Academic research on the social responsibility of startup companies during the pandemic.

References	The positive effect of social responsibility by startups during the pandemic
Xu and Pei, 2020	Startup value
Jiang, 2020	Customer loyalty behaviors, startups’ sense of identity
He et al., 2020	Improve employee resilience
Cui and Guo, 2020	Recognition emotion, consumer trust, startup reputation, product quality
Wang and Qun, 2020	Purchase intention

During the pandemic, fulfilling social responsibilities has had many positive effects on startups. Because the pandemic has presented severe operating conditions, consumers have a greater sense of identity with startups that take their social responsibility seriously (Vethirajan et al., 2020). Consumers perform subjective evaluations of the reliability and social reputation of startups based on this sense of identity (Luo and Bhattacharya, 2006). When a startup has a high degree of social participation, it obtains a higher degree of consumer favorability, which helps to enhance the value image of the startup (Sen and Bhattacharya, 2001). Based on enhancing the social responsibility of startups, responding to consumer satisfaction during the pandemic can effectively improve employee resilience and ultimately form an organic integration of startups’ brand image to achieve the organic unity of startups’ social and economic values (Hussain et al., 2020).

The fulfillment of social responsibilities by startups can effectively enhance their social reputation, brand image, and goodwill among consumers; create economic value; and improve their performance (Kim and Kim, 2020). The social responsibilities performed by startups during the pandemic have positive externalities, transforming their internal startup corporate culture, brand image, and startup reputation into consumers’ deep-level recognition of startups and startup products, thereby forming an “altruistic and self-interested” mechanism. Practicing the social value of startups through philanthropy and other means externally transforms the intrinsic value of startups, effectively constructs the social image of startups, and improves the resilience of employees (Glavas, 2016b), building a startup corporate culture. The timely and effective handling of issues, such as the protection of the legitimate rights and interests of employees, has highlighted the responsibility mission and responsibility of startups during the pandemic, creating startup social responsibility internally. However, the impact of startups’ fulfillment of social responsibility on their performance cannot be achieved overnight; rather, it needs to be carried out gradually (Seivwright and Unsworth, 2016). More importantly, an enterprise’s ability to fulfill its social responsibilities is positively related to its economic strength, and it needs to operate within the bounds of the market economy. The government and the public should allow enterprises to choose a donation method that suits their own economic interests in accordance with the laws of the economy, but the government can donate to enterprises. The scale and direction of the company provide a certain degree of guidance.

## Discussion

### Construction of Social Responsibility and Strategic Choices of Startups During the Pandemic

#### Carroll’s Corporate Social Responsibility Model

**FIGURE 3:**
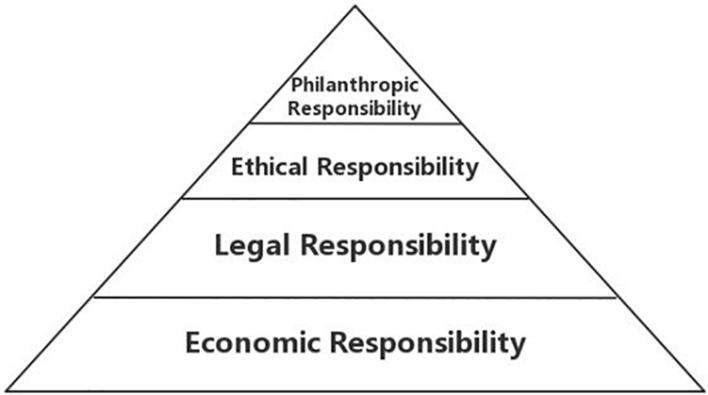
Carroll’s startup CSR pyramid.

Carroll’s definition of CSR is illustrated as a pyramid (shown in Figure 3), which represents economic responsibility, legal responsibility, ethics, and charitable donations. These four elements are progressive. Economic responsibility is at the bottom of the pyramid, confirming that profit is the foundation of everything. Legal responsibility is the second level of the pyramid, indicating that startups need to abide by the law. The law is a social system of right and wrong, and startups must play according to the rules of the game. Rational responsibility is the third level. Startups are ethical and are obliged to conduct activities in ways that are correct, fair, and equal to avoid harm. Philanthropic responsibility is at the top of the pyramid, indicating that startups need to be good corporate citizens who contribute resources to society and improve their quality of life (Carroll, 1979, 1991a).

#### Rethinking the Startup Corporate Social Responsibility Model

**FIGURE 4:**
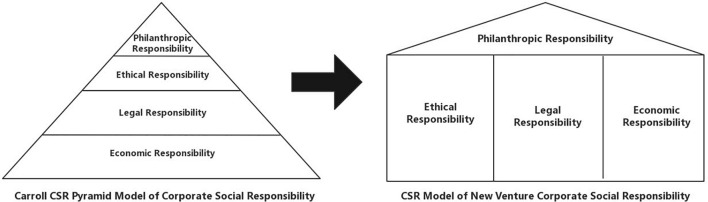
Revised startup CSR responsibility model in response to the pandemic.

The pandemic has not only had a huge impact on human production and lifestyles but has also collided with traditional social moral values, creating unprecedented difficulties in the formation and practice of social responsibility by startups (Jebran and Chen, 2020). In Figure 4, we propose a new four-part CSR model of startups based on Carroll’s original startup CSR model, combined with the SCP analytical framework. This new model adopts the four dimensions of Carroll’s model: economic responsibility, legal responsibility, ethical responsibility, and charitable responsibility. During the normalization stage of the pandemic, companies need to develop a complex principle of ethical, legal, and economic responsibilities. When major changes occur in the external environment, companies themselves need to regroup their social responsibilities to develop a better social enterprise responsibility model with a wide range of social significance and value. We should clarify that the four dimensions are not mutually exclusive and superimposed on each other, but are arranged according to the development sequence of CSR and the relative importance of each dimension.

#### Strategic Choices in Startups’ Corporate Social Responsibility in Response to the Pandemic

At present, the biggest challenges for startups in fulfilling their social responsibilities are ideas and awareness. Some startups are profit oriented, and some consider only short-term benefits. From a short-term perspective, the social responsibility of startups requires investment. For this reason, many startups consider that the gains outweigh the losses and seldom take the initiative toward socially responsible behaviors (Mazutis and Slawinski, 2015). The social responsibility of startups is not only a product of social pressure and legal constraints, but also self-regulatory behavior by startups.

From a strategic perspective, the social responsibility of startups should be a sustainable goal. It is an important strategic orientation for startups to mitigate risks and increase opportunities for profit, rather than a burden created by the pandemic that would reduce the social responsibility of startups. The further growth of startups is truly aided only after their focus shifts to sustainable development (Babu et al., 2020).

In recent years, the social responsibility requirements for startups have increased. Startups use CSR as a strategy to enhance their corporate image. This has led to a continuous increase in the number of startups’ social responsibility behaviors worldwide. These behaviors not only help address local difficulties and problems, but also improve the good image of startups in the long term and ultimately positively impact economic performance (Frederick, 2009). Amid the COVID-19 pandemic, for startups to perform CSR behaviors effectively, they need to consider the social background and characteristics of the external environment comprehensively and to construct appropriate CSR strategies. The core leaders and teams of startups need to return to their original aspirations and rethink the social responsibilities of startups. During periods of huge fluctuation in the external environment, startups need to coordinate the internal and external development of the core team to deal with possible interruptions in products, sales, and after-sales services. New corporate meaning is derived during the pandemic from the problem of stabilizing the production capacity of the entire startup, completing the offline supplies directly connected to online consumers, achieving a normal state of operations at the startup company in a depressed world market environment, and discovering shortcomings in original operations and many other detailed issues (Ahmed et al., 2020). Startups need to think long term, establish a social responsibility organizational system, formulate social responsibility development plans, and evaluate their social responsibility. Every startup company should assume this responsibility from the day it is created. The startup needs to fulfill its commitment to employees, consumers, and partners and to create social value. Only in this way can it achieve sustainable development and better practice during the pandemic, fulfilling its social responsibility.

### Interdependence Between “Economic Man” and “Social Man”

According to Carroll’s startup CSR pyramid model, in a normal period, the social responsibility of startups is hierarchical. For a startup, CSR is ranked from high to low based on its importance, reflecting various CSR values at different levels (Glavas, 2016a). However, when constructing the CSR model during the normalization period of the pandemic, this study pays more attention to the harmonious symbiosis of the attributes of “economic man” and “social man” in CSR, and clearly clarifies the starting point of CSR, to avoid conflicts within the enterprise owing to changes in the external environment.

During the pandemic, society has placed higher expectations on startups to take social responsibility seriously, and the economic and social attributes of startups’ social responsibility have been magnified. The government, the public, and startups have created their own requirements for the social attributes that startups want to reflect from different angles (Zhao et al., 2020). This requires startups to take responsibility when they assume and demonstrate their social responsibilities (Dahlsrud, 2010). There is a clearer understanding of the dual attributes of startups: they are not only an economic organization but also a social organization and an important part of the social fabric. Startups grow out of particular social demand, relying on a certain social environment based on public support. Startups and society should be interdependent and mutually motivating, and should develop simultaneously. Startups need to integrate the external environment and form an integrated development model with society. Only in this way can they maintain their youthful vitality. The harmonious coexistence of the attributes of “economic man” and “social man” in CSR is inseparable from the coordinated assistance of the three responsibilities of ethics, law, and economy.

Therefore, startups need to achieve a harmonious symbiosis of the characteristics of “economic man” and “social man” to fulfill their social responsibility. In the unified structure of the two concepts, they should base themselves more appropriately on actual social conditions and achieve broad applicability. The social responsibility mechanism of startups with vitality in the new era should improve the internal and external norms of their social responsibility, and finally, should achieve a harmonious symbiosis between their social and economic values.

### Parallel Tripartite Economic, Ethical, and Legal Responsibilities

First, during the pandemic, ensuring that startups can operate well is the basis of an important preliminary and material guarantee for startups to assume social responsibility. Conversely, if a startup company is not managed well, it cannot effectively assume its corresponding social responsibilities. However, this does not mean that the function of startups is simply to create profits. We cannot define the social responsibility of startups by the amount of profit they generate, nor can we unilaterally deny startups the ability to earn profits. Evaluating the social responsibility of startups from the perspective of revenue, profit, and their own development would be biased. Startups need to earn profits and develop their potential. They also need to assume and fulfill their social responsibilities in this process. In doing so, the economic responsibility of startups is undoubtedly the basis of an important material guarantee. Startups and shareholders’ profits are realized, and the goals of startups related to mutual benefits with society are irrelevant. The maximization of any one goal is constrained by another goal. The pursuit of profit goals by startups and the pursuit of social goals often mutually influence and restrain each other. Under conditions of mutual restraint, profit goals and social goals require the relative maximization of their respective goals, so that the overall goal of the startups becomes more balanced.

Second, legal responsibility is an inevitable requirement for startups to assume social responsibility (Ostas, 2010). Startups must operate in accordance with the law and abide by labor law, product quality law, consumer rights protection law, tax law, environmental law, etc. Startups need to ensure they do not transgress the legitimate rights and interests of employees, provide inferior products, harm the legitimate rights and interests of consumers, or evade or avoid paying taxes. Tax fraud and environmental harm are violations of both business performance and the social responsibility of startups. The basic prerequisite for the survival and development of startups is to abide by laws and regulations. Only by operating in accordance with the law and fulfilling their minimum social responsibilities will they be recognized and accepted. If they engage in producing and selling fake and shoddy products that damage the interests of other stakeholders, they will be severely sanctioned by the law and condemned by the public.

Finally, enterprises are not just simple profit-making entities, but also exist in the social system as “corporate citizens.” From the perspective of corporate citizenship, at different stages of development, all companies must follow not only external laws and regulations, but also the framework of traditional ethics and morals to form a corporate economic image and a corporate economy (Wu and Sharpe, 2020). Under the normalization phase of the pandemic, we construct a CSR model consisting of three equal responsibilities: corporate economic responsibility, corporate legal responsibility, and corporate ethical responsibility. At the same time, a corporate social responsibility system with realistic significance is developed, so that there are stable and supportive mutual influences and interactions among ethical responsibility, economic responsibility, and charity responsibility, which fundamentally protect the enterprise’s orderly development of social responsibility.

### Charitable Responsibility: Still the Highest Pursuit

Following the CSR model of Carroll, our model prioritizes charitable responsibility during the pandemic for the following three main reasons.

1. Charitable responsibility highlights the external social attributes of startup CSR. Startups and the external added value they generate depend on society. Startups can develop and change by relying on social progress. After startups acquire the corresponding attributes at the social level, they should give back to society in their own way. An important form of giving back is for startups to consciously assume responsibility for charity and to fulfill their social responsibility.

2. Charitable responsibility has core attributes that enhance the inherent humanistic value of a startup’s CSR. If startups want to fulfill their social responsibilities, they need coordinated development in the external environment and internally. Charitable responsibility can drive startups to form an inherent startup culture with correct values and development orientation.

3. Charitable responsibility can better realize sustainable development in the social responsibility of startups based on the dimensions of ethical, legal, and economic responsibility. The core concept of charitable responsibility effectively interacts with ethical, legal, and economic responsibility, and they complement each other in the construction of a more stable startup CSR development model.

When performing their social responsibilities, startups need to develop a social responsibility system with realistic significance under the multilateral correction of laws, ethics, and ethics.

Startups also need to adhere to the core ideas of charitable responsibility, which should be clear and ethical. The mutual influence and role of ethical, legal, and economic responsibility effectively ensure that startups can develop and advance on the correct track and achieve the ultimate goal of driving Chinese startups to fulfill their social responsibilities better.

## Conclusion

### Future Choices for Startups

CSR has shifted from “ignoring people” to being “concerned about people” in theory and practice. Startups must focus on the long-term development of people to better obtain employee support, consumer support, higher brand awareness, and public recognition.

First, startups should prioritize and guarantee the safety and health of their employees. In the process of fulfilling their social responsibilities, startups should pay attention to the real life and psychological situation of employees through various activities and provide multidimensional training for employees. Second, the pandemic has impacted the employment and production methods of startups, such as the replacement of labor by technology in labor-intensive startups, artificial intelligence, and the large-scale application and popularization of unstaffed factories. From a longer-term perspective, startups need to pay more attention to the development of employees, consider the future development trajectory of each employee responsibly, give full play to the intrinsic value and role of each employee, and create sustainable development work for employees’ success. Third, placing importance on human development requires startup companies to use existing technology and resources to address the problem of unbalanced social development and to empower disadvantaged groups and people in underdeveloped areas. After the outbreak of the COVID-19 pandemic, a large number of startups actively assumed social responsibilities; spared no efforts in donating money; organized volunteer activities; helped and supported customers, suppliers, communities, and employees; and ensured the health of stakeholders. In this way, they contributed to China’s battle against the pandemic.

Only by becoming socially responsible can startups gain a position in a fiercely competitive market, truly build their own cultural core, and overcome the challenges of the pandemic to form internal corporate values and concepts that, when combined with external overall market dynamics, build startup teams with core competitiveness, and truly realize the sustainable development of startups. Facing challenges amid the pandemic, startups have looked for opportunities in the crisis, paid attention to the development of people, and focused on the coordination and unity of the value of people and the development of startups (Liu, 2018). This fits Amartya Sen’s view of free development (Navarro, 2000) and is consistent with Marx’s view of the comprehensive and free development of humankind (Chen, 2005). The pandemic gave the core teams of startups and even startups as a whole experience in adapting to a changing market environment, and allowed more investors to observe the value of investment in startups. The wheels of history will not stop turning because of the pandemic, and human progress will not stop because of it. The pandemic will not be the end, but rather the starting point for the development and transformation of startups.

China is now in the stage of normalization after the epidemic. The social responsibility of new startups has changed significantly during the normalization stage. This study analyzes CSR through the SCP analytical framework, which intuitively reflects the social responsibility of startups. As the epidemic conditions develop, countries worldwide will gradually enter the stage of normalization after the epidemic, and the social responsibilities of new startups will also change accordingly. This study provides a forward-looking theory for the development of new startups in the normalization period after the epidemic, a framework, and strategic choices.

## Data Availability Statement

The original contributions presented in the study are included in the article/supplementary material, further inquiries can be directed to the corresponding author.

## Author Contributions

CL and LL described and developed the literature review and the hypothesis. HZ and BL were involved in the data collection process. LL performed the analysis and interpretation of the results and formulated the main conclusion. BL and LL formulated the study limitations and future directions for research. All the authors helped to edit and format the manuscript and contributed equally to this paper.

## Conflict of Interest

The authors declare that the research was conducted in the absence of any commercial or financial relationships that could be construed as a potential conflict of interest.

## Publisher’s Note

All claims expressed in this article are solely those of the authors and do not necessarily represent those of their affiliated organizations, or those of the publisher, the editors and the reviewers. Any product that may be evaluated in this article, or claim that may be made by its manufacturer, is not guaranteed or endorsed by the publisher.
